# Mindful and Intuitive Eating Imagery on Instagram: A Content Analysis

**DOI:** 10.3390/nu14183834

**Published:** 2022-09-16

**Authors:** Johanna K. Hoare, Natalie B. Lister, Sarah P. Garnett, Louise A. Baur, Hiba Jebeile

**Affiliations:** 1 Children’s Hospital Westmead Clinical School, The University of Sydney, Westmead 2145, Australia; 2 Institute of Endocrinology and Diabetes, The Children’s Hospital at Westmead, Westmead 2145, Australia; 3 Kids Research, The Children’s Hospital at Westmead, Westmead 2145, Australia; 4 Weight Management Services, The Children’s Hospital at Westmead, Westmead 2145, Australia

**Keywords:** intuitive eating, mindful eating, mindfulness, nutrition, social media, young adults

## Abstract

Non-dieting approaches, including mindful/intuitive eating, to health improvement are of increasing interest, yet little is known about young adults’ social media exposure to them. Therefore, this study aimed to describe the imagery related to mindful/intuitive eating which is visible to young adult Instagram users. Images categorized under the hashtags ‘mindfuleating’ and ‘intuitiveeating’ were searched in September 2021 using the ‘top posts’ view. Screen captures of 1200 grid-view images per hashtag were used to construct coding frameworks and to determine saturation. Sample sizes for #mindfuleating and #intuitiveeating were 405 and 495 images, respectively. Individual images were coded collaboratively. Almost half of each sample depicted food or drink, of which 50–60% were healthy foods. Approximately 17% were single-person images, of which the majority were young, female adults with healthy weight. Approximately one-third of text suggested credibility through credentials, profession, or evidence. Messaging was similar for both hashtags, encompassing mindful/intuitive eating (~40%), nutrition/eating behaviours (~15%), physical/mental health (~20%), disordered eating (~12%), and body-/self-acceptance (~12%). Differences were observed between hashtags for weight-related concepts (20%/1%) and anti-diet/weight-neutral approaches (10%/35%). The representation on Instagram of mindful and intuitive eating portrays healthy lifestyles without a focus on weight but lacks demographical and body-type diversity. Instagram holds the potential for health professionals to disseminate culturally/demographically inclusive, evidence-based health/nutrition information to youth.

## 1. Introduction

Many young people engage with social media platforms such as Instagram [1,2]. In the U.S., 63% of surveyed youth aged 15–25 years reported using Instagram in 2020 [2]. While some suggest that social media can be a positive educational health and wellbeing resource for young people [3,4], other data show adverse effects such as increased body dissatisfaction and disordered eating [5,6]. Social networking sites are frequently used to search for nutrition-related information [7], and this may inform health behaviour changes [7,8]. Content endorsed by peers, celebrities, and relatable organizations may further influence perceptions. Hence, young people may be particularly vulnerable to making health-related decisions based on digitally available, unregulated information [7]. Inadequate access to appropriate health services and evidence-based information may exacerbate young people’s reliance on digital sources [9,10]. Additionally, some young people with limited health literacy may have difficulty evaluating the credibility of information sources [1,11,12]. Understanding the digital content that young people see online, which may influence their health and wellbeing [10], will facilitate the design of targeted, acceptable, and effective health interventions [7].

The transition from adolescence to adulthood is a period when excess weight changes may occur and when young people are interested in diets [13,14,15,16]. Non-dieting approaches, including mindful and intuitive eating, for health improvement without a focus on weight loss are of increasing interest [17,18,19,20,21]. Mindful eating promotes attentive and purposeful eating experiences focusing on the moment without judgment [22]. Intuitive eating further accounts for emotional eating and cognitive distortion, promoting body acceptance and self-compassion, pleasurable movement, and emphasizing the enjoyment of food without dieting or attaching moral values to foods or eating behaviours [23]. Intuitive eating aims to develop internal awareness of hunger and satiety sensations while engaging, trusting, and acting upon the body’s signals related to eating [24,25].

Mindful eating interventions have effectively reduced some maladaptive eating behaviours, including binge eating and emotional eating [26]. Young people with higher levels of intuitive eating have demonstrated reduced body dissatisfaction [27] and increased wellbeing [28]. In adults, interventions incorporating mindfulness, meditation, and mindful/intuitive eating have resulted in some positive health outcomes such as diet quality [29] and improvement in eating disorders [18]. However, data specific to young people are limited [18,19,30]. While there is the potential for mindful and intuitive eating to improve health outcomes, previous content analyses of Instagram have highlighted concerns about a lack of diversity and promotion of a thin-body ideal [9,31]. Therefore, the aim of this study was to describe imagery related to mindful eating and intuitive eating with high engagement on the image-sharing social media platform Instagram, visible to young people.

## 2. Methods

### 2.1. Study Design

This content analysis explored popular images posted on Instagram under the hashtags ‘mindfuleating’ and ‘intuitiveeating’. We captured images appearing under the ‘top posts’ view, reflecting high engagement by the user community. For each hashtag, we first constructed a coding framework and determined the sample size using a novel method described in Supplementary Materials File S1 [32,33,34,35,36,37,38,39,40]. We then coded the images using the frameworks and examined the data to identify recurring topics.

Instagram employs multiple methods to increase the visibility of user-generated ‘posts’ to intended audiences. For example, the hashtag system categorizes content via user-defined labels. Instagram supports the assignment of up to 30 hashtags per post, making the content visible through various search terms. The top-posts view, available through the mobile application, collates popular content by accounting for the number of users ‘liking’, commenting on, and sharing a specific post. The imagery appearing under this view is additionally based on the user’s prior browsing behaviour, determined by a multifactorial algorithm [35,36]. To minimize an undue influence on the results, Instagram content that was specific for each hashtag was searched using a newly created user account for a young adult aged 21 years (gender not specified). The mobile device was cleared of browsing history prior to each search.

### 2.2. Data Capture

Data were captured on 3 September 2021 for #mindfuleating and on 5 September 2021 for #intuitiveeating. For each hashtag, 1200 images were obtained using a mobile device screen capture function, saved, and numbered sequentially. Included records were single images, and the first images of ‘carousel posts’ that group together multiple images and/or audio–visual material. Video thumbnails and screen captures of autoplay video recordings were excluded.

The grid views, displaying collages of ~15 images per screen (depending on the device), and individual images were captured simultaneously during the searches. The online posts were also ‘saved’ under the new user accounts, facilitating the extraction of additional data (username, number of followers, concurrently assigned hashtags) after the initial image capture. The coding framework development and the sample size determination were based on the grid view images cropped to a 1:1 square aspect. The content was coded based on the individual images. Full-aspect images and the hashtags (within the captions and comments) were saved using screen capture. The username and number of followers were retrieved through the saved posts and captured on a spreadsheet. Data were considered missing and excluded from analysis if, during the time elapsed between the initial search and the extraction of additional data from the saved posts, (1) the user account or post had been deleted and could not be accessed to determine the number of followers and capture hashtags, or (2) the hashtags had been amended such that the original tag (#mindfuleating or #intuitiveeating) was no longer assigned to the post. All data were retrieved from the public domain, with consent from submitting parties not required.

### 2.3. Coding Frameworks and Coding Procedures

**Figure 1:**
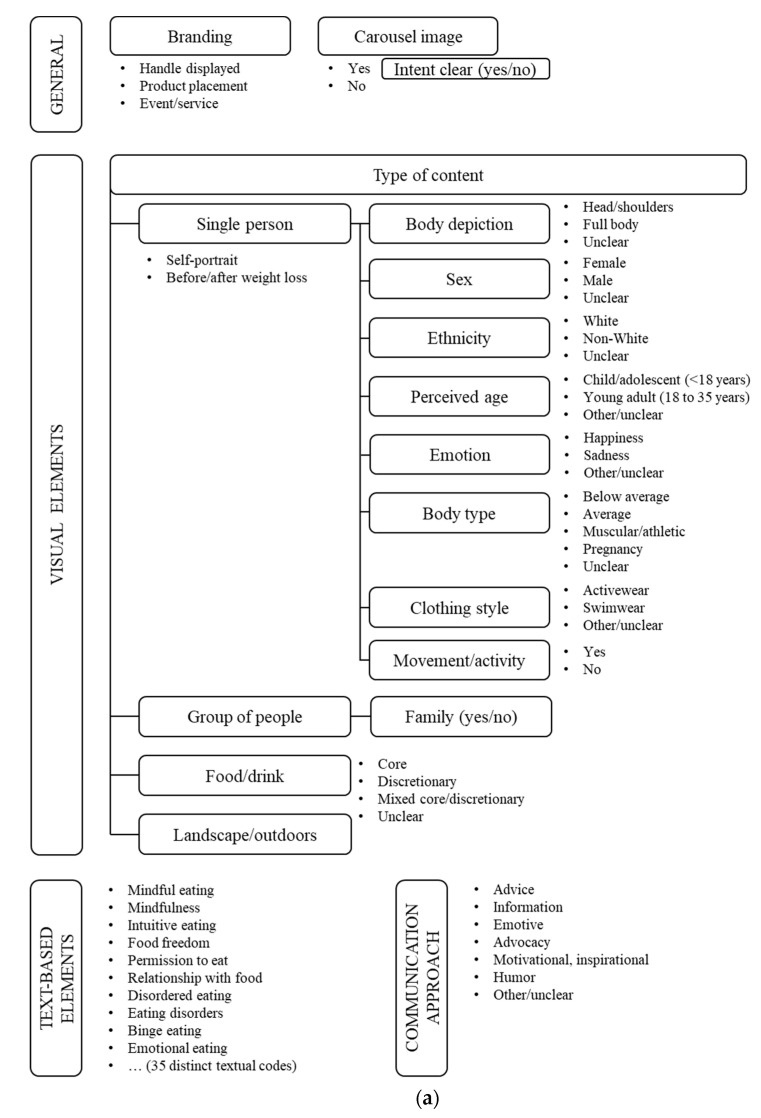

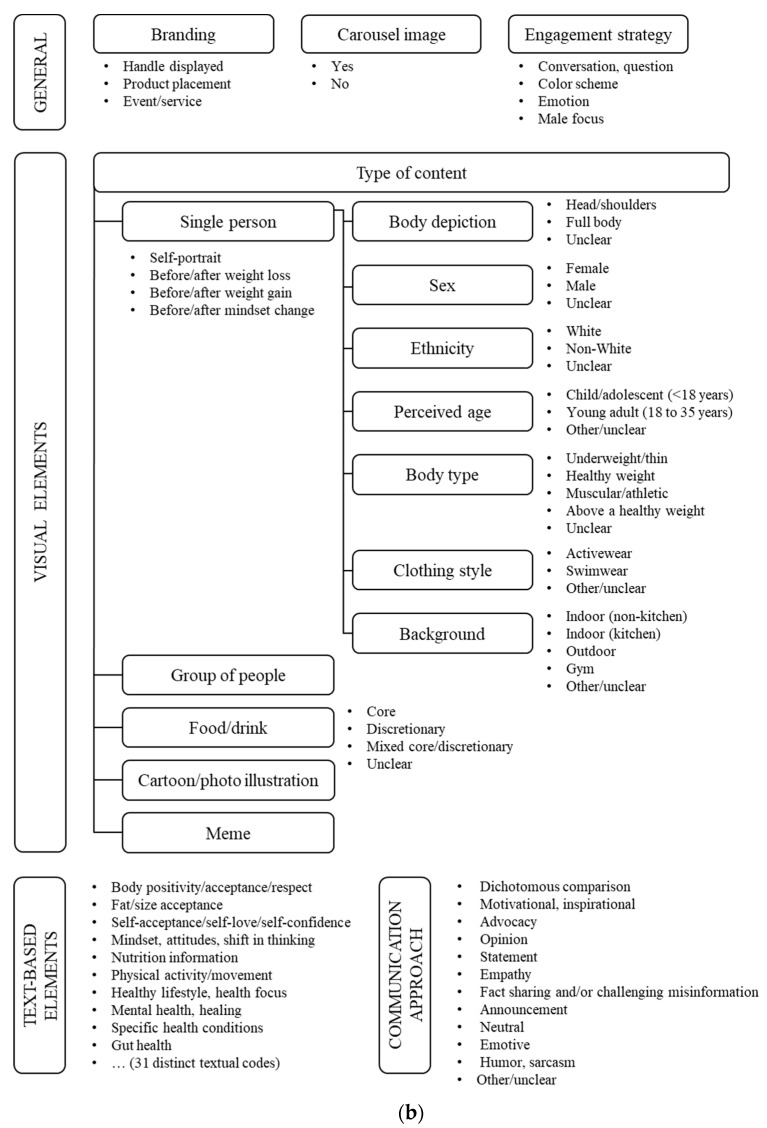
Extracts of the coding frameworks developed for (**a**) #mindfuleating and (**b**) #intuitiveeating.

To develop the coding framework, we initially captured 1200 images from each hashtag. This number was based on previously described methods using sample sizes from 600 [31,41,42] to 1000 or more [37,38,39]. Hashtag-specific coding frameworks were constructed to inform the design of coding instruments using Research Electronic Data Capture (REDCap) software [43] hosted at The University of Sydney, New South Wales, Australia. The frameworks (Figure 1) were constructed based on the visual and textual elements within the image frame, including the graphical representation such as text styles and the use of emojis.

First, two authors (J.K.H. and H.J. for #mindfuleating; J.K.H. and N.B.L. for #intuitiveeating) collaboratively developed a draft coding framework by determining the codes for the first 90 images for each hashtag. Then, the codes were determined independently for increments of 45 images, the results were compared, and conflicts were resolved by discussion. This iterative process continued until new codes were no longer identified, marking data saturation. Textual messages were considered only when they were written in English. The overarching categories within the coding framework were based on the type of content (such as ‘body type’), whereas the codes described the specific content (e.g., ‘thin’, ‘athletic/muscular’). Images of people were categorized for demographical and appearance-related characteristics. Food and drink images were coded as healthy (‘core’) and unhealthy (‘discretionary’) items, determined based on the Australian Dietary Guidelines [44]. The textual categories involved the message content and the communication style.

The coding framework for #mindfuleating (Figure 1, Supplementary Materials Tables S1 and S2) comprised 74 unique codes, with saturation occurring at 405 images. There was one duplicate image which was considered as a separate record, reflecting the viewer’s exposure to the content. The framework for #intuitiveeating (Figure 1, Supplementary Materials Tables S1 and S3) featured 86 codes, with saturation reached at 495 images. The saturation points were used as the sample sizes in this content analysis.

One author (J.K.H.) coded all images independently for the two hashtags. All coding was checked with a second researcher (H.J. for #mindfuleating and N.B.L. for #intuitiveeating). Disagreements were resolved by discussion.

### 2.4. Data Analysis

The frequencies and prevalence of individual codes were calculated, and code combinations of interest (e.g., people with food; self-portraits with a gym background) were described using SPSS^®^ Amos™ 28.0. The image sources were examined separately to determine the number of individual user accounts in the sample, the frequency of occurrence, and the number of followers. The concurrently assigned hashtags were analysed for the most frequently used hashtags, their broad topic areas, and the average number of hashtags assigned to posts.

## 3. Results

### 3.1. General Content

A total of 403 #mindfuleating and 491 #intuitiveeating images met the inclusion criteria (after excluding 2 and 4 video files, respectively) and were coded. Almost half had pictorial elements only, over a quarter contained exclusively textual elements, with the remaining images including both pictorial and textual elements. ‘Carousel posts’ represented 17–18% of each hashtag sample, and almost half of the samples contained branding, most commonly displaying a handle, hashtag, or brand name. Promotion of commercial products was present in less than 5% of each dataset. The textual content under #intuitiveeating was mainly in English, while #mindfuleating featured approximately 30% of non-English textual content, most frequently Spanish. Posts under #intuitiveeating used a range of engagement strategies, with approximately a third written in a conversational tone.

### 3.2. Visual Content

#### 3.2.1. #Mindfuleating

Pictorial elements were identified in 294 of 403 images (73%), with the majority of visual content depicting either food and/or drink (48%, *n* = 192) and/or single persons (18%, *n* = 74) (Table 1, Supplementary Materials Table S1). The food and drink imagery featured core items (62%, *n* = 119) more frequently than discretionary food/drink (21%, *n* = 41), with 23 images showing mixed core/discretionary items. From 74 images of people, women (91%, *n* = 67, of whom half were White, a quarter non-White, and a quarter unclear) featured more than men (7%, *n* = 5, of whom four were White). Young adults (78%, *n* = 58) were depicted most frequently, and most individuals showed their full body (90%, *n* = 66). Only one image compared a person before and after weight loss. Depicted body size was perceived as healthy weight in 67% (*n* = 44) of people, muscular or athletic in 14% (*n* = 9), with 11% (*n* = 7) below healthy weight. There were no images of people with larger bodies. The predominant emotion was happiness (63%, *n* = 46). People were depicted most commonly in casualwear (79%, *n* = 52) or activewear (18%, *n* = 12). Images portraying swimwear or depicting movement/activity were uncommon.

#### 3.2.2. #Intuitiveeating

Of 491 images, 339 (69%) contained pictorial elements. Most of these images were of food and/or drink (45%, *n* = 220) or single persons (16%, *n* = 79) (Table 1, Supplementary Materials Table S1). Healthy (‘core’) items represented half of the food and drink imagery, with discretionary items featuring in a quarter of food images. Of 79 images of individual people, approximately a quarter were depicted with food and/or drink. Individuals were mostly women (95%, *n* = 75, of whom 57 were White) and predominantly young adults (72%, *n* = 57). The majority of individuals showed their full body (91%, *n* = 52), with self-portraits comprising almost a fifth of the images of people (18%, *n* = 14), mostly featuring activewear and a gym/locker room background. There were few before versus after weight loss (*n* = 5) or weight gain (*n* = 2) images. More than half of the people were perceived to have a healthy weight, followed by approximately one-fifth with muscular/athletic bodies, seven people with larger bodies and three people with thin bodies. Clothing style was predominantly casual (81%, *n* = 42) or activewear (17%, *n* = 9).

### 3.3. Textual Content

#### 3.3.1. #Mindfuleating

There were 145 images with textual content in English, summarized in Table 2. More than one-third of these images presented a credibility claim through referring to evidence or providing health-focused professional qualifications or other credentials perceived to reflect expertise (e.g., coach, trainer, therapist). A single image could contain, and be coded for, multiple textual topics. The textual messages related to mindful eating, intuitive eating, mindfulness, relationship with food, permission to eat, and/or food freedom in 37% (*n* = 53) of images with English-language content (Table 2, Supplementary Materials Table S2). The messaging featured in approximately the same 20% proportion included: (1) dietary and nutritional information (coded as specific diets, dietary patterns; eating behaviours; portion sizes; nutrition information; nutrition labelling, *n* = 31); (2) weight-related content (coded as body weight/weight-related, weight loss, dieting, obesity, or bariatric, *n* = 29); (3) messages regarding physical/mental health and wellbeing (coded as healthy lifestyle behaviours/interventions, specific medical condition or health improvement, mental health, or health perception, *n* = 29). Additionally, appearing in approximately 10% of images with English-language content each, the content related to (1) the weight-neutral paradigm (coded as weight-neutral, anti-diet culture, and anti-wellness industry, *n* = 14); (2) disordered eating (coded as disordered eating, eating disorders, binge eating, emotional eating, and/or overeating, *n* = 14); (3) self and the body (coded as body acceptance/respect/image, self-care, self-acceptance, *n* = 14). Half of the messages were presented as advice (*n* = 74), while informational, motivational/inspirational, and emotive messaging each featured in 10–15% of the messages.

#### 3.3.2. #Intuitiveeating

In the #intuitiveeating sample, there were 255 images with textual content in English. These messages were frequently (43%, *n* = 110) presented with a claim of credibility through nutrition- or health-related credentials or job titles (Table 2). Intuitive eating was directly addressed in 19 images (Supplementary Materials Table S3). One-third of the messages related to food freedom, permission to eat, relationship with food, and/or food rules (32%, *n* = 82). Similarly, one-third discussed concepts related to weight-neutral approaches and diet culture (coded as weight-neutral, anti-weight loss, anti-diet, anti-diet culture, anti-fat bias, rejecting beauty ideals, fat/size acceptance, and/or anti-oppression, 35%, *n* = 88). One-fifth addressed physical and mental health (coded as healthy lifestyle/health focus, mental health/healing, specific health conditions, gut health, reproductive health, and/or physical activity/movement, 21%, *n* = 54). Equally, one-fifth related to mindsets, attitudes, shift in thinking, joy, and/or happiness (20% *n* = 52). Additional topic areas, featuring in 10–15% of the textual content each, included eating disorders (coded as disordered eating, eating disorders, overeating, and/or recovery), the body and self (coded as body positivity/body acceptance/body respect/body appreciation, and/or self-acceptance/self-love/self-confidence), and nutrition and eating (coded as nutrition information, eating behaviours/patterns, and/or specific diets). Weight-loss- (*n* = 3) and children/parenting-related topics (*n* = 11) were uncommon. The messaging was presented primarily as an opinion or advice (41%, *n* = 104) or motivational/inspirational (22%, *n* = 55). Emotive language was present in 28 images, while empathy, dichotomous comparison, announcement, advocacy, and humour were used less frequently.

### 3.4. Concurrent Hashtags

From 405 and 495 records for #mindfuleating and #intuitiveeating, information on concurrent hashtags was extracted for 398 and 489 records, respectively (excluding 2 and 4 video recordings, and 2 and 5 records with missing data, respectively). Instagram posts under #mindfuleating and #intuitiveeating were assigned to 19 and 24 concurrent hashtags on average, respectively. The most common concurrently assigned hashtags are summarized in Table 3. Concurrent hashtags were broadly related to (1) food freedom (#foodfreedom, #allfoodsfit); (2) health at every size (#healthateverysize, #haes); (3) self/body acceptance (#selfcare, #selflove, #bodyacceptance); (4) diet culture (#dietculture, #antidiet, #dietculturedropout, #ditchthediet); (5) eating disorder recovery (#edrecovery, #eatingdisorderrecovery); and (6) healthy lifestyle (#nutrition, #healthylifestyle, #healthyeating, #healthyfood, #health).

### 3.5. Source Accounts

Usernames (i.e., image sources) were extracted for 403 and 491 images under #mindfuleating and #intuitiveeating, respectively, originating from 266 and 324 unique user accounts (Supplementary Materials Table S4). The majority (>70%) of accounts appeared only once in each sample, with a small number of accounts posting up to 10 images. Overall, the number of followers ranged from 1000 to 100,000, with a median number of followers under #mindfuleating and #intuitiveeating of 9153 and 12,366, respectively.

## 4. Discussion

This study describes the user-generated content related to mindful and intuitive eating on Instagram. The posts were largely positive and congruent with the key concepts, with the imagery predominantly depicting healthy food and portraying people of average body type. Instagram content related to mindful and intuitive eating appears to be positioned to outline the fundamental concepts of these paradigms [22,23], encourage the adoption of body positivity and self-acceptance, and promote healthy lifestyles. Based on the imagery, the messaging appeared to portray concern over psychosocial wellbeing, through emphasizing individual responsibility for improving health and wellbeing through behaviour and mindset change. Body positivity and permission to eat were frequently presented in the context of diet culture, mainstream beauty ideals, and weight-related discrimination, and some emotive language was identified. Some content was presented from a position of perceived credibility through qualifications or experience. Advocacy for policy action or calling for broader measures [45] appeared uncommon. However, an analysis of the captions (additional text posted with an image) was not conducted, and some underlying meanings may not have been detectable from the image alone. Future thematic analyses and congruence with the underlying paradigms of mindful and intuitive eating will provide additional insights.

Our study revealed a lack of demographic and body diversity in the imagery. There was little portrayal of higher weight, with only two of 495 images under #intuitiveeating perceived to depict larger bodies and no such images appearing under the #mindfuleating imagery. This is consistent with previous analyses, with ‘weightloss’ and ‘fitspiration’ imagery on Instagram found to depict primarily females, frequently in body-accentuating poses [9,31,37]. Another study exploring content tagged as ‘fatspiration’ and ‘healthateverysize’ [46] found the imagery to predominantly represent White women with perceived body size in the intersection of healthy weight and overweight categories. The portrayal of mostly young White females of average body type in this study reflects the lack of diversity on mainstream media and social media platforms. Similar demographic imbalance is evident in the literature [47], where the majority of participants recruited into intuitive eating interventions were White females with mean age 16–51 years. Notably, in one study exploring associations between intuitive eating and dietary intake [48], higher intuitive eating scores were reported in men (48% of participants) than women, highlighting the importance of gender-inclusive and tailored interventions. Hence, the portrayal of a person who would benefit from mindful and intuitive eating may be seen as one-dimensional, raising potential challenges in the context of these approaches being suitable for broader demographic groups and all body sizes and body types.

Our results offer practical implications for future health-intervention efforts. In our study, we found that more than a third of the textual content within a given image was presented with a claim of credibility, with authors frequently identifying as a health professional such as dietitian, nutritionist, doctor, therapist, coach, or counsellor. This is noteworthy since the content available on social media such as Instagram may influence young people’s nutrition-related choices [7,8]. Additionally, it has been suggested that intuitive eating interventions may lend themselves well to self-management [47], which may include online information. In a 2016 study investigating Facebook use for health information among college students (*n* = 351, aged 18–29 years), the participants rated information to be more credible and useful when presented by health professionals, compared with media and peers [49]. Young people, influenced by content endorsed by peers, celebrities, and relatable organizations, may be particularly likely to make health-related decisions based on digitally available information [7]. Future research is required to evaluate how social media users and different demographic groups determine the trustworthiness of online information. For example, such a study might evaluate how young adults with obesity perceive imagery that is non-representative of larger bodies. Future health interventions aimed at young adults should also consider the sources of health-related information that may influence decision making and behaviour changes. Content analyses can support intervention design by highlighting contemporary behavioural drivers and anecdotal sources that may need to be counterbalanced by directing participants to evidence-based information.

Our study has several strengths. The principles of our method are relevant to a range of Instagram searches and social media platforms. We used a customized sample size to summarize the imagery with high engagement to ensure adequate content representation of hashtags that grouped over one million images each. The highly subjective textual content within an image was coded collaboratively. There are also limitations. Instagram is a highly dynamic environment with users commonly modifying the content. A snapshot of top posts is currently unsupported by data-scraping software, and a time lag related to manual data extraction resulted in some missing data. Our coding also did not account for the username, tagged locations, captions, or comments. However, these data may influence perceptions; for example, posts made by expert sources or tagging foodservice venues might have a different influence on their viewers than those without these elements. Additionally, it is unknown what type of user engagement shaped our top-posts sample or who usually views content categorized under our chosen hashtags, and how young people may perceive the content.

## 5. Conclusions

Our findings demonstrate that the overall depiction of mindful and intuitive eating paradigms on Instagram appears to emphasize average-bodied White young female adults and healthy lifestyles without a focus on weight. The representation of males, or diverse body types, ages, and racial/ethnic populations was modest, and this lack of demographical and body-type diversity may reduce the acceptability of mindful and intuitive eating in broader populations. Instagram holds the potential for health professionals to disseminate culturally and demographically inclusive, evidence-based health promotion and nutrition information to young people.

## Figures and Tables

**Table 1:** Summary of pictorial content visible under #mindfuleating and #intuitiveeating, as percentage of total sample.

Visual Element	#Mindfuleating % (*n* = 403) *	#Intuitiveeating % (*n* = 491) **
Images containing pictorial elements (photograph, cartoon, illustration) with/without text	73% (294)	69% (339)
Food and/or drink, of which	48% (192)	45% (220)
Core	62% (119)	50% (110)
Discretionary	21% (41)	26% (56)
Mixed	12% (23)	16% (36)
Unclear	5% (9)	8% (18)
Single person, of whom	18% (74)	16% (79)
Female	91% (67)	95% (75)
Young adult	78% (58)	72% (57)
White	51% (38)	72% (57)
Healthy weight	59% (44)	35% (28)
Other	22% (87)	14% (67)

* *n* = 403 (of total sample 405), excluding 2 video recordings. ** *n* = 491 (of total sample 495), excluding 4 video recordings.

**Table 2:** Summary of textual content categorized under #mindfuleating and #intuitiveeating on Instagram, as percentages of images with textual messaging in English.

Message Group	Codes	#Mindful-Eating (*n* = 145)	#Intuitive-Eating (*n* = 255)
Perceived credibility	Credibility alluded to by credentials, job title, and/or evidence	37% (53)	43% (110)
Mindful/intuitive eating	Mindful eating (ME), mindfulness (ME), intuitive eating, food freedom, permission to eat, relationship with food, food rules (IE)	37% (53)	40% (101)
Nutrition, eating behaviours	Nutrition information, eating behaviours, specific diets/dietary patterns, portion sizes (ME), nutrition labelling (ME)	21% (31)	11% (28)
Physical and mental health	Healthy lifestyle behaviours and interventions, specific medical condition or health improvement, mental health, health focus (IE) health perception (ME), healing (IE), gut health (IE), reproductive health (IE)	20% (29)	21% (54)
Disordered eating, eating disorders	Disordered eating, eating disorders, overeating, binge eating (ME), emotional eating (ME), recovery (IE)	10% (14)	15% (39)
Body-/self-acceptance	Body acceptance/body respect, body image, self-care, self-acceptance, self-confidence	10% (14)	15% (38)
Weight-related concepts	Weight loss, Body weight/weight-related (ME), dieting (ME), obesity (ME), bariatric (ME)	20% (29)	1% (3)
Anti-diet and weight-neutral approaches	Weight-neutral, anti-weight loss, anti-diet culture, anti-diet (IE), anti-fat bias (IE), fat/size acceptance (IE), anti-wellness industry (ME), rejecting beauty ideals (IE), anti-oppression (IE)	10% (14)	35% (88)
Mindset and attitudes	Mindset, attitudes, shift in thinking, happiness, joy	n/a	20% (52)

IE—#intuitiveeating only; ME—#mindfuleating only.

**Table 3:** Thirty-five most common concurrently assigned hashtags in content categorized under #mindfuleating and #intuitiveeating on Instagram, by frequency.

	#Mindfuleating	Frequency (*n* = 398) *	#Intuitiveeating	Frequency (*n* = 489) **
1	intuitiveeating	112	foodfreedom	186
2	mindfulness	86	haes	150
3	foodfreedom	69	antidiet	140
4	nutrition	60	edrecovery	121
5	healthylifestyle	54	healthateverysize	108
6	dietculture	43	eatingdisorderrecovery	104
7	edrecovery	36	nutrition	96
8	allfoodsfit	35	allfoodsfit	94
9	healthateverysize	35	dietculture	92
10	selfcare	34	dietculturedropout	85
11	antidiet	33	healthylifestyle	77
12	haes	32	ditchthediet	73
13	healthyeating	31	bodyacceptance	72
14	selflove	31	healthyfood	72
15	health	30	selflove	72
16	plantbased	30	dietsdontwork	68
17	wellness	30	bodypositive	67
18	ditchthediet	29	health	61
19	bingeeating	28	selfcare	61
20	mindful	27	bodyimage	59
21	weightlossjourney	27	mentalhealth	59
22	emotionaleating	26	bodypositivity	58
23	vegan	26	intuitiveeatingjourney	58
24	foodie	24	disorderedeating	56
25	mindset	24	nondiet	56
26	nondiet	24	bingeeatingrecovery	51
27	fitness	23	dietculturesucks	50
28	nourishnotpunish	23	healthyeating	50
29	dietculturedropout	22	edwarrior	47
30	healthyfood	22	antidietculture	46
31	dietsdontwork	21	mindfuleating	46
32	dietitian	20	foodisfuel	45
33	healthy	20	bingeeating	44
34	mindfulliving	20	intuitiveeatingofficial	44
35	nondietapproach	20	emotionaleating	43

* *n* = 398 (of total sample 405), excluding 2 video recordings and 5 records with missing data. ** *n* = 489 (of total sample 495), excluding 4 video recordings and 2 records with missing data.

## Data Availability

The data presented in this study are available in Table 1, Table 2 and Table 3 and Supplementary Materials Tables S1–S4.

## Supplementary Materials

The following supporting information can be downloaded at: https://www.mdpi.com/article/10.3390/nu14183834/s1, Table S1: General characteristics and visual elements for Instagram content categorized under #mindfuleating and #intuitiveeating, by hashtag and code, Table S2: Textual messaging and communication styles for Instagram content categorized under #mindfuleating, by hashtag and code, Table S3: Textual messaging, communication styles, and engagement strategies for Instagram content categorized under #intuitiveeating, by hashtag and code, Table S4: Summary of image source account characteristics in Instagram content categorized under #mindfuleating and #intuitiveeating, by frequency and number of followers, File S1: A novel method to determine a custom sample size for image-based Instagram content analysis. References [32,33,34,35,36,37,38,39,40] are cited in the Supplementary Materials.
