# Supplementary material for: Mindful and Intuitive Eating Imagery on Instagram: A Content Analysis
Source: Nutrients. 2022 Sep 16;14(18):3834. doi: 10.3390/nu14183834 (PMC9502653; doi:10.3390/nu14183834)
Supplement: Supplementary file 1 [file nutrients-14-03834-s001.zip › nutrients-1908076 Supplementary file 1 - Novel method to determine custom sample size v3.pdf]

Supplementary File S1: A novel method to determine a custom sample size for image-based Instagram content analysis

Methods

Method Development

We adapted our method from grounded theory [32] and thematic analysis [33], used in qualitative research, to inductively develop the coding frameworks and to determine the number of images required to reach data saturation. The iterative sampling of images facilitated the identification of new concepts and representations of ideas by Instagram users. This approach also counteracted the possible impact of the authors' analytic preconceptions of the topic areas [34].

Study Design

Customized sample sizes were addressed in the context of *mindful eating*, promoting being present in the eating experience, and *intuitive eating*, rejecting dieting mentality and food-related moral judgements, and advocating for body respect and joyful movement. Mindful and intuitive eating are used in weight-neutral approaches, with emerging literature investigating their use in younger audiences. Therefore, we created two new Instagram user accounts for a young adult (aged 21 years, gender not specified), each used to search content tagged as either '#mindfuleating' or '#intuitiveeating'.

Each search was performed using the 'top posts' view, available through Instagram mobile application, to simulate high engagement imagery tailored to individual user profiles. This functionality arranges content into a user-specific ranked feed instead of a chronological order [35]. To maximize the relevance for a given user, the content is customized and

prioritized by an algorithm that uses multi-factorial machine learning [36]. Hence, we utilized new user accounts and mobile devices cleared of browsing history.

Data Extraction

Informed by previous studies with large sample sizes [37-39] and to ensure adequate data to reach saturation, 1200 images under each of the two hashtags were captured in July 2021. Source account information, image captions, location tags, concurrent hashtags, and user comments were not included.

Coding Frameworks and Data Saturation

The coding frameworks were developed based on single-image posts and the first images in a series of images (‘carousel posts’), containing image and/or text-based elements, with video content excluded. A separate framework was constructed for each hashtag using an inductive approach [40]. First, two authors collaboratively reviewed the first 90 images, corresponding to viewing six full screens of feed content on a smartphone displaying 15 images per screen. The categories were based on the type of content such as perceived demographic characteristics (‘sex’, ‘ethnicity’, ‘age’). The codes captured the detailed pictorial content (for example ‘female’, ‘White’, ‘young adult’) and the topics represented in textual elements. Consensus was reached through discussion. Next, the draft framework was iteratively developed by two authors independently reviewing and coding the visual content in increments of 45 images. Following each iteration, coding was compared, disagreements were resolved through discussion, and the framework was revised to reflect the dataset as a whole. This process was repeated until no new codes emerged and data saturation was reached. The final increment of 45 images with no new codes emerging was included in the sample size.

Method Reliability

Seven weeks after the initial data captures (September 2021), new data were captured to evaluate the reliability of the method over time. Using the same method and new user accounts, new datasets of 1200 images were extracted from each hashtag. The same researchers then repeated the process of developing the coding frameworks and determining the sample sizes. For each hashtag, the number of codes and the sample sizes were compared between the two time points. The change over time in the proportions of image-based versus textual codes was also evaluated.

Results

Coding Frameworks and Data Saturation

The coding framework constructed for #mindfuleating at baseline and seven weeks comprised 63 and 74 distinct codes, with data saturation occurring at 360 and 405 images, respectively. The framework developed for #intuitiveeating comprised 83 and 86 codes, with saturation reached at 450 and 495 images, respectively. Across all datasets (two hashtags over two time points), the proportion of image-based codes ranged from 34% to 44%, while text-based codes ranged from 49% to 59%, with general codes (related to for example branding and carousel posts) applicable to both types. The results are summarized in **Table 1**.

	**#mindfuleating**	**#intuitiveeating**
**Dataset 1 (week 0)**		
- saturation (number of images)	360	450
- codes (n)	63	83
-% general (n)	6% (4)	7% (6)
-% image-based (n)	44% (28)	43% (36)
-% text-based (n)	49% (31)	49% (41)
**Dataset 2 (week 7)**		
- saturation (number of images)	405	495
- codes (n)	74	86
-% general (n)	*7% (5)*	*14% (12)*
-% image-based (n)	*34% (25)*	*34% (29)*

-% text-based (n)	*59% (44)*	*52% (45)*
**% Change (n) within hashtag over time**		
- saturation	+12.5% (45)	+10% (45)
- codes	+17% (11)	+4% (3)

Table 1: The number of images required to reach saturation in content categorized under #mindfuleating and #intuitiveeating on Instagram, the number and types of distinct codes, and variability between the two time points

Method Reliability

Both hashtags demonstrated an increase in the number of images required to reach data saturation (≥10%) over seven weeks. Similarly, there was an increase in the number of codes determined for the coding frameworks. For both hashtags, the number of image-based codes decreased and the number of text-based codes increased over time (Table 1).

References

32. Charmaz, K. *Constructing Grounded Theory: A Practical Guide through Qualitative Analysis*; Sage: London, UK, 2006, 1–208.

33. Braun, V.; Clarke, V.; Hayfield, N.; Terry, G. Thematic Analysis. In *Handbook of Research Methods in Health Social Sciences*; Liamputtong, P., Ed.; Springer: Singapore, 2019; pp. 843–860.

34. Braun, V.; Clarke, V. Using Thematic Analysis in Psychology. *Qual. Res. Psychol.* **2006**, 3, 77–101.

35. Barnhart, B. How to Survive (and Outsmart) the Instagram Algorithm. Available online: https://sproutsocial.com/insights/instagram-algorithm/ (accessed on 22 April 2022).

36. Warren, J. This Is How the Instagram Algorithm Works in 2021. Available online: https://later.com/blog/how-instagram-algorithm-works/ (accessed on 22 April 2022).

37. Deighton-Smith, N.; Bell, B.T. Objectifying fitness: A content and thematic analysis of #fitspiration images on social media. *Psychol. Pop. Media Cult*. **2018**, 7, 467–483. https://doi.org/10.1037/ppm0000143.

38. Cherian, R.; Westbrook, M.; Ramo, D.; Sarkar, U. Representations of codeine misuse on instagram: Content analysis. *JMIR Public Health Surveill*. **2018**, 4, e22.

39. Laestadius, L.I.; Wahl, M.M.; Pokhrel, P.; Cho, Y.I. From apple to werewolf: A content analysis of marketing for e-liquids on Instagram. *Addict. Behav*. **2019**, 91, 119–127. https://doi.org/10.1016/j.addbeh.2018.09.008.

40. Thomas, D.R. A general inductive approach for analyzing qualitative evaluation data. *Am. J. Eval*. **2006**, 27, 237–246.
