# Supplementary material for: Mindful and Intuitive Eating Imagery on Instagram: A Content Analysis
Source: Nutrients. 2022 Sep 16;14(18):3834. doi: 10.3390/nu14183834 (PMC9502653; doi:10.3390/nu14183834)
Supplement: Supplementary file 1 [file nutrients-14-03834-s001.zip › nutrients-1908076 Supplementary tables S1 to S4.pdf]

Supplementary Table S1: General characteristics and visual elements for Instagram content categorized under #mindfuleating and #intuitiveeating, by hashtag and code

**Code**	**Code/Definition**	**#mindfuleating (*n* = 403)*<br>% (*n*)**	**#intuitiveeating (*n* =491)**<br>% (*n*)**
**IMAGE CHARACTERISTICS**			
Visual elements total	Images containing pictorial elements (photograph, cartoon, illustration) with/without text	73% (294)	69% (339)
Visual + textual elements	Images containing both pictorial and textual elements	25% (99)	24% (118)
Textual elements total	Images containing textual elements with/without pictorial elements, of which<br>*-English*<br>*-non-English*<br>*-Mixed*	52% (208)<br>*- 69% (143)*<br>*- 30% (63)*<br>*- 1% (2)*	55% (270)<br>*- 94% (254)*<br>*- 6% (15)*<br>*- <1% (1)*
Carousel post	The captured image is the first item in a series of images/video recordings posted together as a carousel post<br>*- Intent is perceived as clear based on the first image, of carousel posts that contain text in English on the first image (n=24)*	17% (69)<br><br>*-4% (15)*	18% (88)<br><br>*-n/a*
Branding	Image contains branding of any type	47% (190)	49% (240)
Brand visible	Textual display of an Instagram handle, visible hashtags or a brand, such as a business/account name, or displaying a logo	43% (174)	44% (215)
Product placement	Promotion of a commercial product	4% (15)	5% (23)
Event/service	Promotion of, or quoting an event or a service	<1% (3)	1% (6)
**CHARACTERISTICS OF INDIVIDUALS**			
Single person	Portrayal of single persons (including self-portraits [IE] and before vs. after images), of images containing visual elements, of which<br>*- Female*<br>*- Male*<br>*- Unclear*<br>*-Self-portrait (taken by the person depicted in the image)*<br>*-Self-portrait with a gym background*	18% (74)<br><br>*- 91% (67)*<br>*- 7% (5)*<br>*- 3% (2)*<br>*- n/a*<br>*- n/a*	16% (79)<br><br>*- 96% (75)*<br>*- 4% (3)*<br>*- 0% (0)*<br>*- 18% (14)*<br>*- 5% (4)*
	Before versus after weight loss: two images of the same person, with visibly decreased adiposity in the second image	<1% (1)	1% (5)
	Before vs after weight gain: two images of the same person, with visibly increased adiposity in the second image	n/a	<1% (2)

Code	Code/Definition	#mindfuleating (*n* = 403)* % (*n*)	#intuitiveeating (*n* =491)** % (*n*)
	Before vs after mindset change: two images of the same person, depicted before and after a shift in perceptions (e.g. portraying a difference in mindset, attitudes or thinking, through mental state, emotion, setting or other visible cue)	n/a	<1% (1)
Perceived age	Excludes images of people before/after mindset change [IE]		
	Young adult (18 to 35 years)	14% (58)	12% (57)
	Child/adolescent (less than 18 years)	1% (4)	1% (6)
	Other/unclear: more than 35 years, or unable to determine	3% (12)	3% (15)
Ethnicity	Excludes images of people before/after mindset change [IE]		
	White	9% (38)	12% (57)
	Non-White	5% (19)	1% (6)
	Unable to determine, based on available options	4% (17)	3% (15)
Identifiable emotion	Excludes images of people before versus after a change		
	Happiness (either portrayed or triggering the emotion, such as a person smiling/laughing, engaging in a pleasurable activity or a romantic portrayal)	11% (46)	n/a
	Sadness (either portrayed or triggering the emotion)	<1% (1)	n/a
	Other/unclear	6% (26)	n/a
Body depiction	Excludes self-portraits and before versus after images		
	Head/shoulders, including any part of the arm below the elbow	2% (7)	1% (5)
	Full body, portraying the body below the shoulder level	16% (66)	11% (52)
Clothing type	Includes full body images portraying a single individual, excluding before versus after images and images depicting head/shoulder only		
	Activewear: attire suitable for performing physical activity	3% (12)	2% (9)
	Swimwear, with/without other clothing items present	<1% (2)	<1% (1)
	Other/unclear: Clothing type other than available options, including casualwear, business/formal attire, or unable to determine	13% (52)	9% (42)
Body type	Includes full body Images, excludes before versus after images. Body type was not determined where the angle of the photograph or clothing obstructed the view.		
	Average or medium frame with moderate level of body fat	11% (44)	6% (28)
	Athletic/muscular with visible/high-level definition of muscle; depicting body building or high level sport performance requiring above average fitness	2% (9)	2% (10)
	Thin, slight frame with little-to-no visible fat	2% (7)	<1% (3)
	Larger body with excess body fat	n/a	1% (7)

**Code**	**Code/Definition**	**#mindfuleating (*n* = 403)*<br>% (*n*)**	**#intuitiveeating (*n* =491)**<br>% (*n*)**
	Pregnancy	<1% (1)	n/a
	Other/unclear	1% (5)	<1% (4)
Movement/activity	Portrayal of a person engaging in physical activity or movement, based on full body Images (excluding before vs. after images)	<1% (3)	n/a
Background/setting			
	Indoor (non-kitchen): home, workplace, retail, food service venues, changerooms	n/a	4% (21)
	Kitchen (residential/commercial): showing food preparation, food preparation areas, and/or kitchen appliances	n/a	<1% (2)
	Outdoor: landscape/scenery, or open air setting (e.g. the natural environment, garden, outdoor food service)	n/a	4% (20)
	Gym: exercise equipment shown (not a generic dressing room or bathroom)	n/a	<1% (1)
	Other/unclear: background is blurry, or the main focus of image occupies majority of the image with inadequate detail to determine the background, or other than available options	n/a	2% (8)
Group of people	Portrayal of more than one person, of images containing visual elements<br>*- Family depicted: child/ren with one or two adults (not including romantic couples without children)*	4% (17)<br>*- <1% (2)*	2% (9)<br>*- n/a*
**FOOD/DRINK IMAGERY**	Images portraying food and/or drink, of images containing visual elements, of which<br>*- Core food/drink****<br>*- Discretionary food/drink****<br>*- Mixed core and discretionary items****<br>*- Unclear*	48% (192)<br><br>*- 62% (119)*<br>*- 21% (41)*<br>*- 12% (23)*<br>*- 5% (9)*	45% (220)<br><br>*- 50% (110)*<br>*- 26% (56)*<br>*- 16% (36)*<br>*- 8% (18)*
**OTHER IMAGERY**			
Cartoon/photo illustration	Photographic or cartoon illustrations, present to clarify or highlight a message	n/a	10% (51)
Landscape/outdoor	Landscape/scenery, or the background/setting of the image is outdoors (open air, including the natural environment, garden, and outdoor food service)	5.1% (15)	n/a
Meme	An idea/behavior/style designed to be shared through the Internet/social media for entertainment/amusement	n/a	<1% (2)
Other/unclear	Visual content other than available options, or unable to determine	18.7% (55)^	1 % (5)
**PEOPLE DEPICTED WITH FOOD/DRINK**			
Single person	Images of individual persons that include food/drink, of which	6% (26)	4% (21)

Code	Code/Definition	#mindfuleating (*n* = 403)* % (*n*)	#intuitiveeating (*n* =491)** % (*n*)
	*- Core food/drink****<br>*- Discretionary food/drink****<br>*- Mixed core and discretionary items****<br>*- Unclear*	*- 58% (15)*<br>*- 15% (4)*<br>*- 8% (2)*<br>*- 19% (5)*	*- 19% (4)*<br>*- 29% (6)*<br>*- 19% (4)*<br>*- 33% (7)*
Group of people	Images of groups of people that include food/drink	1% (6)	<1% (2)

[IE], included in #intuitiveeating coding framework only; n/a, not included in the coding framework for a given hashtag;

* *n* = 403 (of total sample 405), excluding two video recordings

** *n* = 491 (of total sample 495), excluding four video recordings

*** based on the Australian Dietary Guidelines

^ includes illustrations

Supplementary Table S2: Textual messaging and communication styles for Instagram content categorized under #mindfuleating, by hashtag and code

**Code**	**Definition**	**Sample *n* = 403*<br>% (*n*)**
Credibility of information	Citing literature or official documents/guidelines, or the source self-identifying (via Instagram handle or post content) as a health professional such as a dietitian or a doctor, or other party with perceived qualifications, expertise or authority to provide credible information, including non-specific credentials such as "therapy", "coach", "counseling", "trainer", "nutrition/ist". Excluding credibility based on account names that are not visible in the post.	13% (53)
Mindful eating	Content related to: being present and using all senses to engage in the eating experience; observing the body's hunger/satiety cues; trusting and honoring one's body to regulate appetite and eating (when/where/how much); refraining from making moral judgements about food/eating; endorsing weight-neutral non-restrictive approach to eating	4% (16)
Mindfulness	Information/advice related to mindfulness or mindfulness meditation	1% (4)
Intuitive eating	Content related to: observing the body's hunger/satiety cues, cognitive distortion, emotional eating. Rejects diet culture. Promotes harmonious relationship with food and the body. Follows the 10 principles of Intuitive Eating (Tribole & Resch): (1) reject the diet mentality, (2) honor your hunger, (3) make peace with food, (4) challenge the food police, (5) respect your fullness, (6) discover the satisfaction factor, (7) honor your feelings without using food, (8) respect your body, (9) exercise—feel the difference, and (10) honor your health.	<1% (2)
Food freedom	Information/advice related to being confident and in control of one's food choices; consumption of all foods without guilt/negative associations; promoting all foods being equal with no moral judgement.	1% (5)
Permission to eat	Information/advice related to the removal of conditional rules pertaining to eating, such as exercising to "earn" calories	1% (6)
Relationship with food	Content related to the thinking and attitudes around food and eating, excluding specific content related to other specified categories (such as intuitive eating, permission to eat, food freedom)	5% (20)
Specific diets, dietary patterns	Information/advice related to a specific dietary pattern such as dietary control of blood glucose, ketogenic diets, fasting, low carbohydrate, mediterranean, non-evidence-based fad diets	2% (10)
Eating behaviors	Information/advice related to eating behaviors, i.e. the timing and frequency of meals; eating-related customs and traditions	1% (6)
Portion sizes	Information/advice related to amount of food to consume	<1% (1)
Nutrition information	Information/advice related to nutritional requirements or recommendations; the nutritional content, functional properties, or macronutrient composition of foods; food preparation advice	3% (12)
Nutrition labelling	Information/advice related to nutrition labelling and provision of written information on the nutritional content	<1% (2)
Body weight, weight-related	Content related to body weight, or weight-related issues	2% (8)
Weight loss	Content related to weight loss, or portrayal of higher body weight/larger bodies as undesirable	3% (14)
Dieting	Information/advice related to dietary intake for the purpose of weight loss	1% (4)
Obesity	Content related to physical obesity/adiposity, or the societal norms related to obesity	<1% (2)
Bariatric	Information/advice related to bariatric surgery, such as pre-surgery weight loss	<1% (1)
Health perception	Information/advice related to one's views on health and health behaviors	1% (5)

Code	Definition	Sample *n* = 403*<br>% (*n*)
Healthy lifestyle behaviors and interventions	Objective/subjective information/advice related to dietary intake, physical activity, sleep, stress-reduction and other lifestyle behaviors	3% (11)
Specific medical condition or health improvement	Information/advice pertaining to specific health conditions such as diabetes, ADHD, PCOS; fertility/pregnancy/erectile dysfunction; inflammation, reflux	2% (9)
Mental health	Information/advice related to specific mental health conditions (depression, anxiety), stress, overwhelm	1% (4)
Weight-neutral	Posts opposing weight loss, and/or promoting weight-neutral concepts (such as health at every size or non-diet)	<1% (1)
Anti-diet culture	Content related to undesirable weight-related societal attitudes, expressing opposing views on diet culture, or perceived stigmatisation	2% (10)
Anti-wellness industry	Content expressing opposing views on wellness industry, such as financial gain from promoting products/approaches seen as undesirable	<1% (3)
Disordered eating	Information/advice related to disordered eating behaviors and patterns, such as food rules pertaining to allowed foods and how/how much/when they should be eaten, following habits/patterns over needs/desires, exercising moral judgement towards food (good/bad foods), feeling one must 'earn' food through exercise; cognitive distortions and disordered thinking pertaining to eating and foods	<1% (1)
Eating disorders	Information/advice related to fear of weight gain, and specific eating disorders (excluding binge eating), recovery, or ED in general	<1% (2)
Binge eating	Information/advice related to binge eating	1% (5)
Emotional eating	Information/advice related to emotional eating, including stress eating using food to cope with a variety of distressing emotions, including anxiety and depression; comfort eating, and boredom eating	<1% (3)
Overeating	Information/advice related to excessive eating, i.e. consuming more energy than physiologically needed, consuming large portions causing a person to feel uncomfortably full. May describe a discrete incident of eating too much (such as during holidays) or a more persistent pattern of overeating	<1% (3)
Body acceptance, body respect	Information/advice related to and/or aiming to improve attitudes on one's body, including body acceptance, body positivity, and body respect (by self/others); addressing body dissatisfaction	2% (9)
Body image	Information/advice related to body image, either enhancing knowledge or aiming to improve body image and reduce body dissatisfaction; addressing attitudes pertaining to the physical body	<1% (2)
Self-care, self-acceptance	Information/advice related to, and/or aiming to improve or maintain positive self-concept including self-compassion, self-acceptance, self-worth, self-esteem, self-image, self-love	<1% (3)
Quote – non-specific	A quote, unrelated to a specific category	2% (10)
Children	Content related to children, paediatric issues, and/or parenting	2% (7)
Pandemic	Content providing information/advice/opinion on facts, beliefs and/or behaviors related to the Covid-19 pandemic	<1% (1)
Other	Textual content not applicable for any other specified category	1% (5)
Unclear	Unable to determine the textual content	<1% (2)
**COMMUNICATION STYLE**	Textual elements in English or mixed English and non-English	145
Advice	Expression of an opinion, a belief, view or judgement by an individual or a group/majority of people	18% (74)

Code	Definition	Sample *n* = 403*<br>% (*n*)
Information	A definite or clear expression of fact-based information	5% (22)
Emotive	Deliberate choice of words to influence or to elicit emotion, used to attract attention, enhance the message or to elicit a response such as a behavior change.	4% (16)
Advocacy	Advocating for change that is sincerely perceived as positive, by defending/promoting a cause on behalf of other people	<1% (2)
Motivational, inspirational	Content designed to motivate, inspire or stimulate interest in something, aiming for change in attitudes or behavior	6% (23)
Humour	Texts with a funny, amusing or comic quality	<1% (3)
Other/unclear	Unrelated, or unable to determine the communication approach	5% (21)

* #mindfuleating *n* = 403 (of total sample 405, excluding two video recordings), of which 145 images containing textual messages in English

Supplementary Table S3: Textual messaging, communication styles and engagement strategies for Instagram content categorized under #intuitiveeating, by hashtag and code

Code	Definition	Sample *n* = 491* % (*n*)
Evidence-based, credibility	Messages presenting evidence-based information or alluding to credible sources of information	22% (110)
Intuitive eating	Mindful eating: Content related to: being present and using all senses to engage in the eating experience; observing the body's hunger/satiety cues; trusting and honoring one's body to regulate appetite and eating (when/where/how much); refraining from making moral judgements about food/eating; endorsing weight-neutral non-restrictive approach to eating; mindfulness meditation. Intuitive eating: Content related to: observing the body's hunger/satiety cues, cognitive distortion, emotional eating. Rejects diet culture. Promotes harmonious relationship with food and the body. Follows the 10 principles of Intuitive Eating (Tribole & Resch): (1) reject the diet mentality, (2) honor your hunger, (3) make peace with food, (4) challenge the food police, (5) respect your fullness, (6) discover the satisfaction factor, (7) honor your feelings without using food, (8) respect your body, (9) exercise—feel the difference, and (10) honor your health.	4% (19)
Food freedom, permission to eat	Information/advice related to being confident and in control of one's food choices; consumption of all foods without guilt/negative associations; promoting all foods being equal with no moral judgement; Posts verbally granting permission to eat particular foods or serving sizes	8% (40)
Relationship with food	Content related to the thinking and attitudes around food and eating, excluding specific content related to other specified categories (such as food rules, food freedom)	5% (26)
Food rules	Information/advice related to rules pertaining to allowed foods and how/how much/when they should be eaten, following habits/patterns over needs/desires, exercising moral judgement towards food (good/bad foods), feeling one must 'earn' food through exercise	3% (16)
Anti-diet, anti-diet culture	Content related to undesirable weight-related societal attitudes, expressing opposing views on diet culture, diet industry or perceived stigmatisation	8% (41)
Weight-neutral, anti-weight loss	Posts opposing weight loss, and/or promoting weight-neutral concepts (such as health at every size or non-diet)	5% (23)
Anti-fat bias	Content challenging fat bias and stigmatisation of individuals with obesity	2% (9)
Rejecting beauty ideals	Content highlighting assumptions/perceptions about beauty, and/or offering alternative perceptions	1% (7)
Fat acceptance, size acceptance	Information/advice/opinion pertaining to concepts related to fat/size acceptance movement	<1% (4)
Anti-oppression	Content opposing the perceived oppression experienced by minority groups. Posts aiming to debunk myths, or science/mainstream views	<1% (4)
Healthy lifestyle, health focus	Information/advice pertaining to physical health, determinants of health, healthy lifestyle behaviors, with the intent of improving health outcomes	4% (19)
Mental health, healing	Information/advice related to specific mental health conditions (depression, anxiety), stress, overwhelm; content related to healing from a specified or unspecified condition/mental state	3% (13)

Code	Definition	Sample *n* = 491* % (*n*)
Specific health condition related information and/or advice	Content related to specific health conditions such as diabetes, ADHD, PCOS, hypothalamic amenorrhea, menopause	2% (10)
Gut health	Information/advice related to gut/digestive health/well-being and/or gastrointestinal diseases/conditions	<1% (2)
Reproductive health	Information/advice/opinion on facts, beliefs and/or behaviors related to fertility or pregnancy	1% (5)
Physical activity or movement related information and/or advice	Information/advice intending to encourage physical activity, exercise, movement	1% (5)
Mindset, attitudes, shift in thinking	Content challenging one's perception or views on a particular topic; encouraging the reader to adopt a new perspective	9% (43)
Happiness, joy	Content related to positive emotions such as happiness and joy	2% (9)
Disordered eating, eating disorders	Content specific to disordered eating behaviors and/or eating disorders. Information/advice related to binge eating, fear of weight gain, and specific eating disorders or ED in general; Stress eating using food to cope with a variety of distressing emotions, including anxiety and depression; Comfort eating, and boredom eating	5% (25)
Recovery	Recovery-related information or advice related to eating disorders, hypothalamic amenorrhea, and other health conditions	2% (11)
Overeating	Information/advice related to excessive eating, i.e. consuming more energy than physiologically needed, consuming large portions causing a person to feel uncomfortably full. May describe a discrete incident of eating too much (such as during holidays) or a more persistent pattern of overeating	<1% (3)
Body positivity, body acceptance, body respect, body appreciation	Content related to attitudes on the physical body, including body positivity, body acceptance, body image. Posts addressing boundaries/body respect by self and others; posts providing information/advice and/or aiming to improve body positivity/acceptance and/or addressing body dissatisfaction	4% (22)
Self-acceptance, self-love, self-confidence	Content related to self-compassion/self-acceptance or positive self-image . Posts providing information/advice and/or aiming to improve or maintain positive self-concept inlcuding self-compassion, self-acceptance, self-worth, self-esteem, self-image, self-love	3% (16)
Nutrition information	Information/advice pertaining to nutrition, with the intent of improving health outcomes or nutritional knowledge. Information/advice related to nutritional requirements or recommendations; the nutritional content, functional properties, or macronutrient composition of foods; food preparation advice	3% (15)
Eating behaviors, eating patterns	Content related to eating behaviors or eating patterns, i.e. the timing and frequency of meals; eating-related customs and traditions	2% (10)
Specific diet related information/advice	Information/advice related to a specific dietary pattern such as dietary control of blood glucose, ketogenic diets, fasting, low carbohydrate, non-evidence-based fad diets	<1% (3)
Child/parent related information and/or advice	Information/advice related to children, paediatric issues, and/or parenting	2% (11)
Weight loss related information/advice	Information/advice pertaining to weight loss, with the intent to motivate/enable individuals to lose weight	<1% (3)
Ethics, values, integrity	Content discussing or challenging the moral basis of promoting an idea or a product	<1% (3)

Code	Definition	Sample *n* = 491*<br>% (*n*)
Covid-19 pandemic-related	Posts making any type of comment or reference related to the Covid-19 pandemic. Content providing information/advice/opinion on facts, beliefs and/or behaviors related to the Covid-19 pandemic	0
Other	Textual content not applicable to available options	3% (14)
**COMMUNICATION STYLE**	Textual elements in English or mixed English and non-English (n=255)	
Opinion	Expression of a belief, view or judgement by an individual or a group/majority of people, or offering personal/professional advice; a view or judgement formed about something, not necessarily based on fact or knowledge; a statement of advice by an expert on a professional matter, or offering personal/anecdotal advice	21% (104)
Motivational, inspirational	Content designed to motivate, inspire or stimulate interest in something, aiming for change in attitudes or behavior. Use of a positive/encouraging tone, aiming to stimulate interest in/enthusiasm for a cause/action/behavior/beliefs	11% (55)
Emotive	Deliberate choice of words to influence or to elicit emotion, used to attract attention, enhance the message or to elicit a response such as a behavior change. Positive or negative overt expressions or subtle connotations, such as emphasizing/exaggerating words/expressions ; Use of nicknames, superlatives, or provoking language	6% (28)
Empathy	Posts written to express understanding, compassion, empathy or solidarity to the reader's circumstances or emotions. Expressions of understanding, compassion, empathy, solidarity; encouraging gratitude; providing assurance	4% (22)
Dichotomous comparison	Content presented as two completely opposing ideas/things, such as truth/myth, true/false, yes/no, fair/unfair, agree/disagree etc.	4% (18)
Announcement	Posts aiming to capture attention in order to provide advice or information perceived important for the reader to know. Using alert words to capture the reader's attention, such as Fact, Reminder, PSA/Public service announcement, FYI, BREAKING; OR "I don't know (OR IDK) who needs to hear this, but…" or " Just so that you know…"	3% (17)
Advocacy	Advocating for change that is sincerely perceived as positive, by defending/promoting a cause on behalf of other people; Promoting/supporting people, belief, cause/ideology/paradigm/movement; guideline/policy/recommendation that is perceived to enhance people's welfare/rights/dignity, and/or positively influence health/quality of life, OR advocating against instances that contradict the above	3% (13)
Humour, sarcasm	Texts with a funny, amusing/comic or sarcastic quality. Use of humour or sarcasm/irony to convey a message, express an opinion or to mock or convey contempt	3% (13)
Fact sharing and/or challenging misinformation	Expression of information perceived to be true, for the purpose of correcting misconceptions or misinfiormation. Sharing of perceived facts to increase knowledge or understanding, and aiming to correct misconceptions or misinformation, such as Myth/Truth.	2% (11)
Statement	A definite or clear expression of fact-based information	2% (10)
Neutral	Deliberate choice of words to convey message in a neutral tone. Messaging that keeps emotion out as much as possible, through careful choice of words and sentence structure. Content presented in a neutral manner, not trying to influence the position of the reader on the matter	2% (9)
Other/unclear	Unable to determine, or not applicable to available options. Includes commercial interests and promotions.	4% (20)
**ENGAGEMENT STRATEGY**	Strategies used by the image as a whole to engage audiences	
Conversation, invitation, question, encouragement	Post written in a manner that aims to increase engagement, such as in a conversational or letter style; sharing of personal experiences; written to the reader (you/your/we/our); invitations or questions, encouraging the reader to take action or reframe	30% (145)

**Code**	**Definition**	**Sample *n* = 491* % (*n*)**
	mindset/attitudes; use of arrow, link, or wording "to find out more" or similar, directing reader to further content; encouraging the reader to comment	
Tone – Color scheme	Images portraying a color scheme or black/white tones, of which<br>*- Pink/pastel (overall appearance (text, image and/or background) dominant in pink, purple or pastel colors)*<br>*- Earthy (overall appearance dominant in earthy tones such as shades of green, brown, orange, or yellow)*<br>*- Black/white (monochrome or grayscale black/white)*<br>*- Other (color scheme other than defined options)*	26% (128)<br>*- 45% (58)*<br>*- 23% (29)*<br>*- 31% (40)*<br>*- <1% (1)*
Tone – Emotion	Images portraying an identifiable emotion, of which<br>*- Positive (Content portraying or triggering positive emotions such as happiness, content, delight, joy; Image portraying person smiling/laughing, engaging in a pleasurable activity)*<br>*- Negative (Content portraying or triggering negative emotions such as anger, frustration, sadness, despair)*	15.1% (74)<br>*- 81% (60)*<br>*-19% (14)*
Male focus	Content specific to male audiences; images depicting majority of male individuals; text specific or relevant to male audiences only	0.6% (3)

* #intuitiveeating *n* = 491 (of total sample 495, excluding four video recordings), of which 255 images containing textual messages in English

Supplementary Table S4: Summary of image source account characteristics in Instagram content categorized under #mindfuleating and #intuitiveeating, by frequency and number of followers

	**#mindfuleating (*n* = 403)***	**#intuitiveeating (*n* = 491)****
Frequency of posts in the sample<br>1<br>2-4<br>>5	Number of accounts (%)<br>203 (76%)<br>53 (20%)<br>10 [5 to 10 posts each] (4%)	Number of accounts (%)<br>232 (72%)<br>83 (26%)<br>9 [5 to 7 posts each] (3%)
Number of followers of accounts owning >5 posts, range	293 - 107,639	3,295 - 232,043
Number of unique accounts posting content (number of items in the sample)	266 (403)	324 (491)
Number of followers<br><br>≤ 1000<br>>1,000 to 10,000<br>>10,000 to 100,000<br>>100,000<br>Average<br>Median<br>Range	Number of user accounts, by number of followers<br>7<br>131<br>115<br>13<br>22,592<br>9,153<br>291 to 292,443	Number of user accounts, by number of followers<br>6<br>138<br>152<br>28<br>34,583<br>12,366<br>371 to 1,033,289

* *n* = 403 (of total sample 405), excluding two video recordings

** *n* = 491 (of total sample 495), excluding four video recordings
